# Evaluating the effectiveness of HOCl application on odor reduction and earthworm population growth during vermicomposting of food waste employing *Eisenia fetida*

**DOI:** 10.1371/journal.pone.0226229

**Published:** 2019-12-19

**Authors:** Chanwoo Kim, Younggu Her, Yooan Kim, Chanhoon Jung, Hangkyo Lim, Kyo Suh

**Affiliations:** 1 Graduate School of International Agricultural Technology, Seoul National University, Pyeonchang-gun, Gangwon, Republic of Korea; 2 Department of Agricultural and Biological Engineering, Tropical Research and Education Center, Institute of Food and Agricultural Sciences, University of Florida, Homestead, Florida, United States of America; 3 Interdisciplinary Program in Agricultural and Forest Meteorology, Seoul National University, Seoul, Republic of Korea; 4 Department of Biology, Notre Dame of Maryland University, Baltimore, Maryland, United States of America; 5 Institute of Green Bio Science & Technology, Seoul National University, Gangwon, Republic of Korea; Gifu University, JAPAN

## Abstract

Vermicomposting has been recommended as an eco-friendly method to transform organic waste into nutrient resources with minimum energy input. However, odor and pest issues associated with this method limit the use of vermicomposting, especially in indoor conditions. This study evaluated the effectiveness of applying hypochlorous acid (HOCl) to deodorize the vermicomposting process and improve the breeding environment for earthworms (*Eisenia fetida*). The deodorization performance of HOCl was compared by measuring the amount of ammonia (NH_3_) and amine (R-NH_2_) released from the decaying process of two types of food waste: HOCl-treated (HTW) waste and non-treated waste (NTW). The total and individual weights of earthworms in the waste treated with HOCl was measured to evaluate the impact on earthworm reproduction after applying HOCl. The results showed that HOCl application could reduce NH_3_ by 40% and R-NH_2_ by 80%, and increase the earthworm population size and total weight by up to 29% and 92%, respectively, compared to the control group. These results suggest that HOCl application is potentially an efficient method to control the odor and to boost earthworm reproduction and thus facilitate vermicomposting for improved food waste treatment and environmental quality.

## Introduction

Sustainable methods for processing food waste have received considerable attention as global food waste has rapidly increased over the past few decades [1]. In South Korea, food waste per capita has increased by more than 14.3% in the last 10 years [2, 3]. Thus, eco-friendly food waste treatment is critical for improved environmental quality. However, only a few countries process food waste separately from other types of waste. In many countries, food waste is disposed of with other landfill waste and/or incinerated through dehydration and combustion using supplementary fuel. However, incineration is relatively expensive and generates harmful gases [4]. Thus, decomposing food waste in landfills is still the preferred solution, even though organic materials and leachate from landfill food waste can contaminate the soil and water and cause environmental problems.

Graff (1974) [5] demonstrated that vermicomposting to process food waste using earthworms is a potentially effective and efficient method to treat food waste. Vermicomposting is recognized as an eco-friendly process because it does not require direct energy input [6]. Several studies have investigated the potential of vermicomposting to process not only food waste but also sludge and livestock manure [1, 7–10]. In addition to breaking down waste, research has demonstrated that vermicomposting could promote plant nutrient uptake and soil microbial activity, and improve soil texture and quality [11–15]. Earthworm activity in the soil could improve soil aggregation and structure, and vermicompost application could improve microbial diversity and populations [16–19]. However, this method has been limited because of the long processing time, space demands, and potential odor and pest issues, especially in indoor conditions. The vermicomposting process requires an extended time to grow the earthworms and requires adequate space to handle the mass of food waste. In addition, the odor and pests created in the process can be a challenge for small-scale, indoor, food waste treatment facilities [20].

Although several vermicomposting studies have focused on the efficiency of growing worms and processing organic waste [6, 21–23], few studies have focused on the odor problem associated with vermicomposting. Mao and Tsai (2006)’s study was one of the first to examine how to efficiently eliminate or control the odor from food waste composting using multiple engineering methods [24]. Edwards and Arancon (2010) later demonstrated how odor from vermicomposting could be controlled using a breeding box [20]. However, the applicability of their method is limited. Whereas a sealed lid can control the odor from breeding bins, opening the lid for moisture control and feed supply diffuses the odor. To date, follow-up studies have not been conducted to improve the efficiency and applicability of their breeding box experiment to reduce odor.

Another method is the application of hypochlorous acid (HOCl), which is a weak acid known to have antimicrobial effects [25, 26]. Many studies have confirmed the sterilizing function of HOCl and demonstrated its effectiveness in alleviating odor [25, 27–30]. At the same concentration, HOCl has an 80 times greater antibacterial effect than that of hypochlorite ion (ClO^-^) [31]. Cai (2016) also demonstrated that HOCl has an antiviral mechanism and thus can be used to regulate viral infections [32]. In 1998, in the United States, the Environmental Protection Agency (EPA) approved HOCI for its safety as a high-level disinfectant [33], and in 2000, the Food and Drug Administration (FDA) approved it as a harmless preservative for a saline solution [34]. These studies and administrative actions have demonstrated the potential of HOCl as an agent for odor and pest control in vermicomposting.

Majlessi et al. (2012) showed that vermicomposting of food waste using earthworms could stabilize the vermicomposting process for plant growth by comparing a seed germination index, C/N ratio, and other stability and maturity indices [35]. Moreover, certain additives to the vermicomposting may promote the vermicomposting efficiency through fast growth of earthworms and thus overcome some of the obstacles of vermicomposting. Zarrabi et al. (2018) also found that vermicomposting using zeolite positively influenced the growth pattern of earthworms to improve the quality of vermicomposting [36]. Previous studies, however, have mainly focused on the sterilizing function and effectiveness of odor control of HOCl. No known studies have investigated the effect of HOCl on both the vermicomposting process and earthworm growth.

The purpose of this study was to evaluate the implications of HOCl application on the food waste vermicomposting process to eliminate the odor and increase earthworm growth. This study tested three vermicomposting conditions with different HOCl applications for moisture control and feed preparation. The test started with three groups with the same earthworm weight and population size. Changes in the three groups were measured after the experimental period (60 days). Odor reduction was quantified by measuring the concentrations of ammonia and amine emitted from the food waste throughout the composting process. The results were statistically analyzed and compared with those of other relevant studies.

## Materials and methods

**Fig 1:**
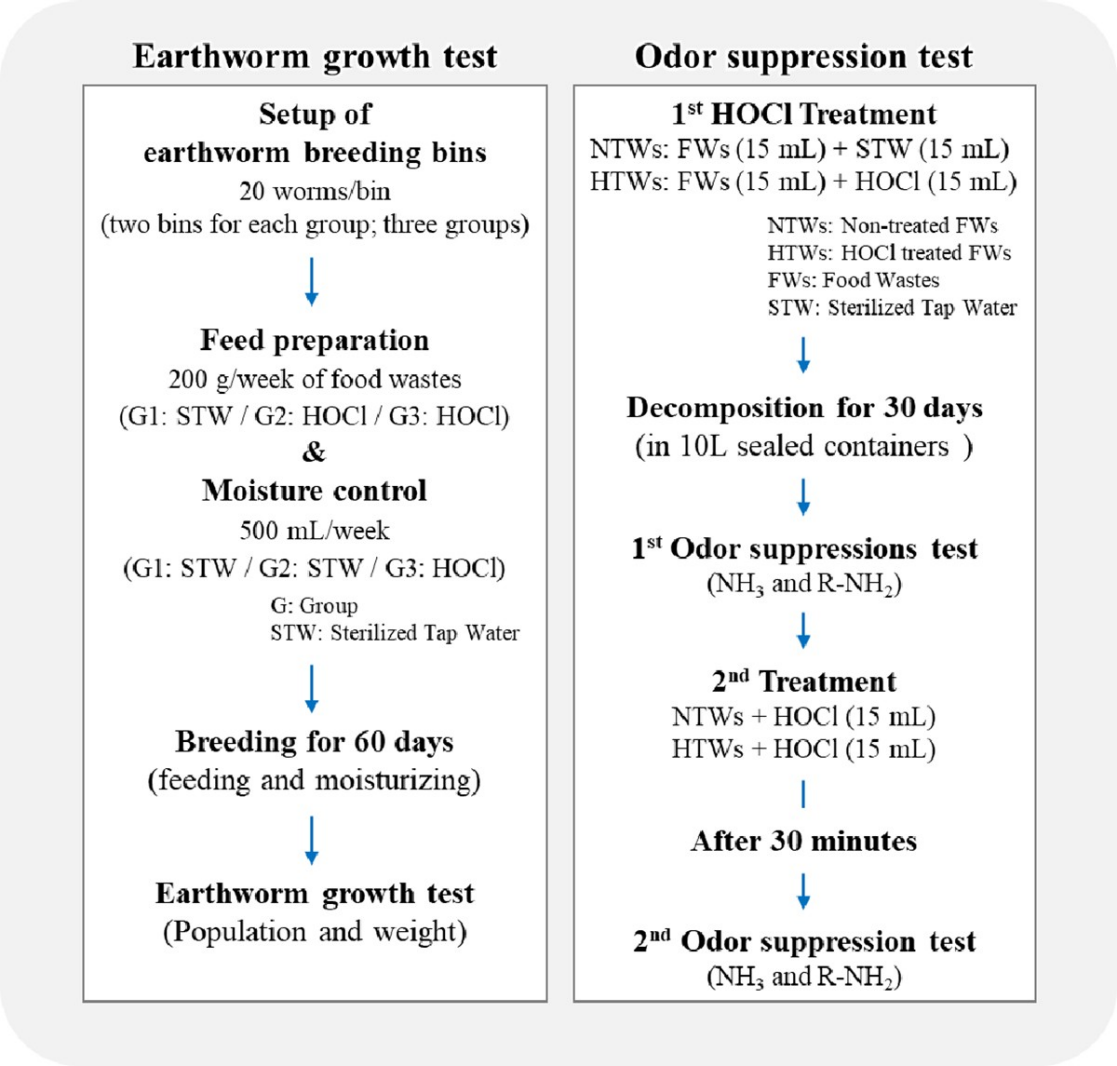
Overall procedure of two independent experiments implemented in this study.

Two independent experiments were designed and conducted to quantify and isolate the effects of HOCl application on earthworm population growth and odor suppression (Fig 1).

### Earthworm and food waste

Earthworms, *Eisenia fetida (*Family Lumbricidae*)*, used in this study can adapt to decaying organic materials and grow well in indoor and laboratory conditions [20, 37]. This species is widely used for vermicomposting of various industrial organic wastes because of their ability to convert organic matter into biomass, which has a high market value [20]. Earthworms for this study were bought from Sekyoung Farm, which is a retailer of earthworms in South Korea.

The earthworm feed consisted of fruit including bananas, apples, watermelons, and melons as well as food waste from a cafeteria in Seoul National University, which was collected each week over the 60-day period. Since salinity in food waste can limit the growth of earthworms, the collected food waste from the cafeteria was fermented for three days to reduce the salinity [38].

### HOCl application in feed preparation

Earthworms could easily adapt because the fermentation process also decayed the food waste. In the earthworm population growth test, 500 mL of HOCl was mixed into the food waste for feed preparation in group 2 before feeding it to the earthworms. In group 3, HOCl was used for both moisture control and feed preparation. As the mass density of food waste widely varies depending on the type of waste, this study determined the amount of HOCl to be applied considering the volume and weight of food waste to be treated. For moisture control, additional HOCl was directly sprayed onto the bedding surface to maintain the proper soil humidity inside the bin: between 50% and 70%.

The HOCl concentration and acidity were in the range of 40 to 50 ppm and pH 5.5 to 6.0, respectively. These amounts met the criteria of slightly acidic electrolyzed water (SAEW), a concentration of 10 to 80 ppm, and acidity pH of 5.0 to 6.5 [39, 40].

### Earthworm population growth test

**Fig 2:**
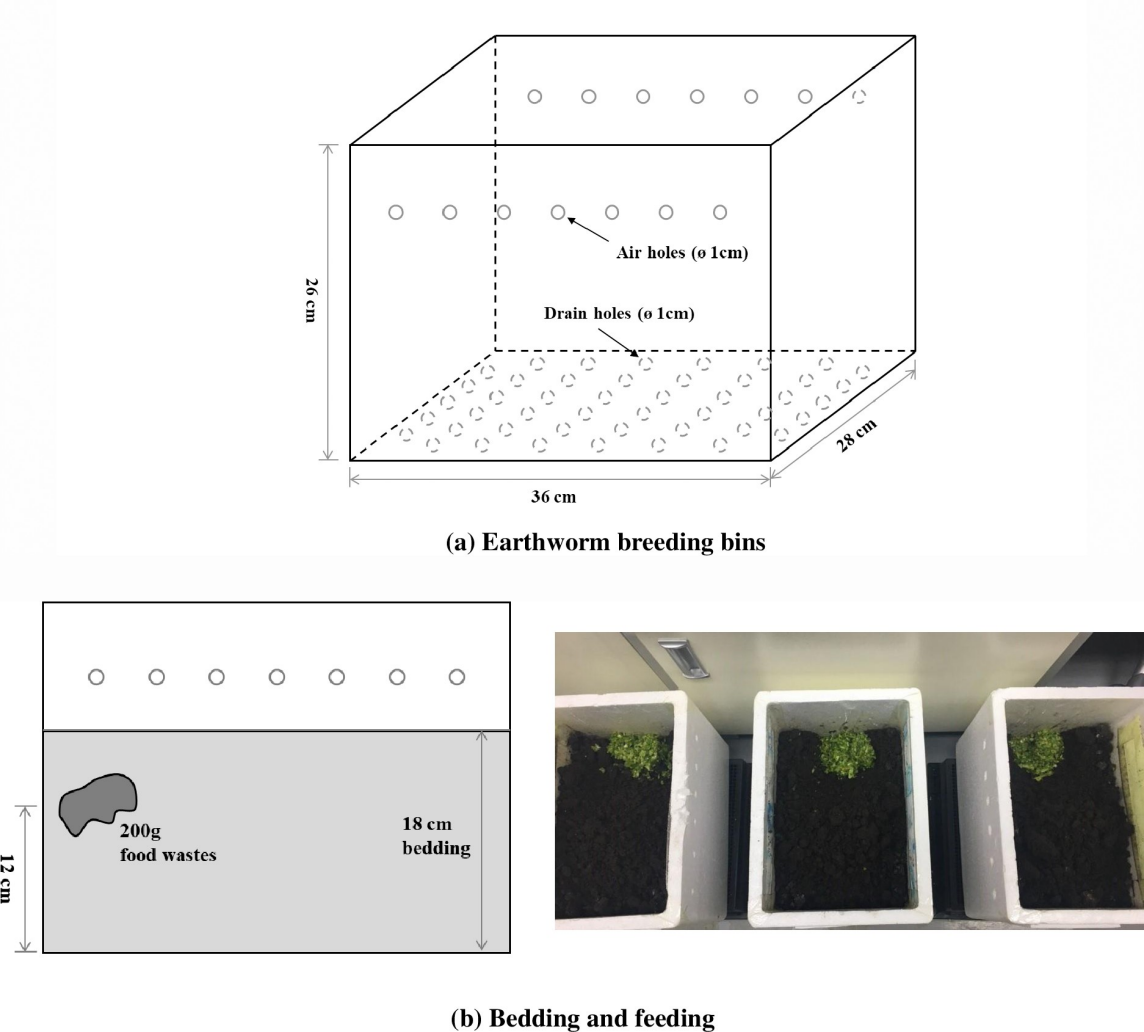
Design of breeding bins with a bedding and feeding plan for 60 days of vermicomposting: (a) represents the design of the earthworm breeding bins. (b) displays the depth of the bedding and the location of feeding (food wastes).

The earthworms were bred separately in six Styrofoam bins with an internal size of L 36 × D 26 × H 28 cm (Fig 2A). Two bins were assigned to each group. Each bin had seven breathing holes with a 1-cm diameter which were located 20 cm above the bottom of each two walls and 45 drainage holes with a 1-cm diameter on the bottom. Each bin was filled with earthworms and 1,250 g compost from the earthworm retailer, which created an 18-cm deep compost layer to provide bedding for the worms (Fig 2B).

To test the effectiveness of the HOCl application, HOCl was applied to the soil for feed preparation and moisture control. This study applied 500 mL of HOCl or sterilized tap water corresponding to the weight of food waste (of 200 g). Sterilized tap water (500 mL) on the water supply was used for moisture control for groups 1 and 2. Group 1 was the control group without any HOCl application with only sterilized tap water (500 mL) for moisture control. For group 2, HOCl (500 mL) was applied to the food waste for feed preparation before feeding it to the earthworms and sterilized tap water (500 mL) was used for moisture control. For group 3, only HOCl was used for both moisture control and feed preparation. In other words, in group 3 bins, all the conditions for feed preparation with HOCl were the same as for group 2, but an additional 500 mL of HOCl was sprayed onto the bedding in the bins during the experimental period for moisture control.

Twenty earthworms were placed in each bin for the three groups; each earthworm weighed 0.5 g, on average, with a standard deviation of 0.2 g. The weight of each earthworm was measured according to the “dump and hand sort technique” method proposed by Appelhof and Olszewski (2017) [41]. Food waste was added to each experimental bin in the designated location after preparing the feed using HOCl every week for 60 days (Fig 2B). The 60-day experimental period was predetermined following the active reproduction duration provided from Warman and AngLopez [6].

### Odor suppression test

Ammonia (NH_3_) and amine (R-NH_2_) gases are the major components and causes of the bad smell generated in the vermicomposting process. Food waste samples were treated by applying 15 mL of HOCl, denoted as HOCl-treated food waste (HTW). HTW and non-treated food waste (NTW) of 15 mL were prepared and fermented in 10 L sealed containers for 30 days at room temperatures between 24°C and 26°C and relative humidity levels between 38% and 49%.

The concentrations of NH_3_ and R-NH_2_ in the containers were measured to quantify the level of odor created from the composting process using a gas detection tube, Gastec GV-50ps. This study replicated the concentrations two times. The gas detection tube consists of a gas sampling pump and a gas detector tube, and gas concentrations can be scaled by reading the color change layer that responds to the concentrations. Details of the gas detection tube method can be found in Thompson and Lotter’s (2014) study [42].

**Fig 3:**
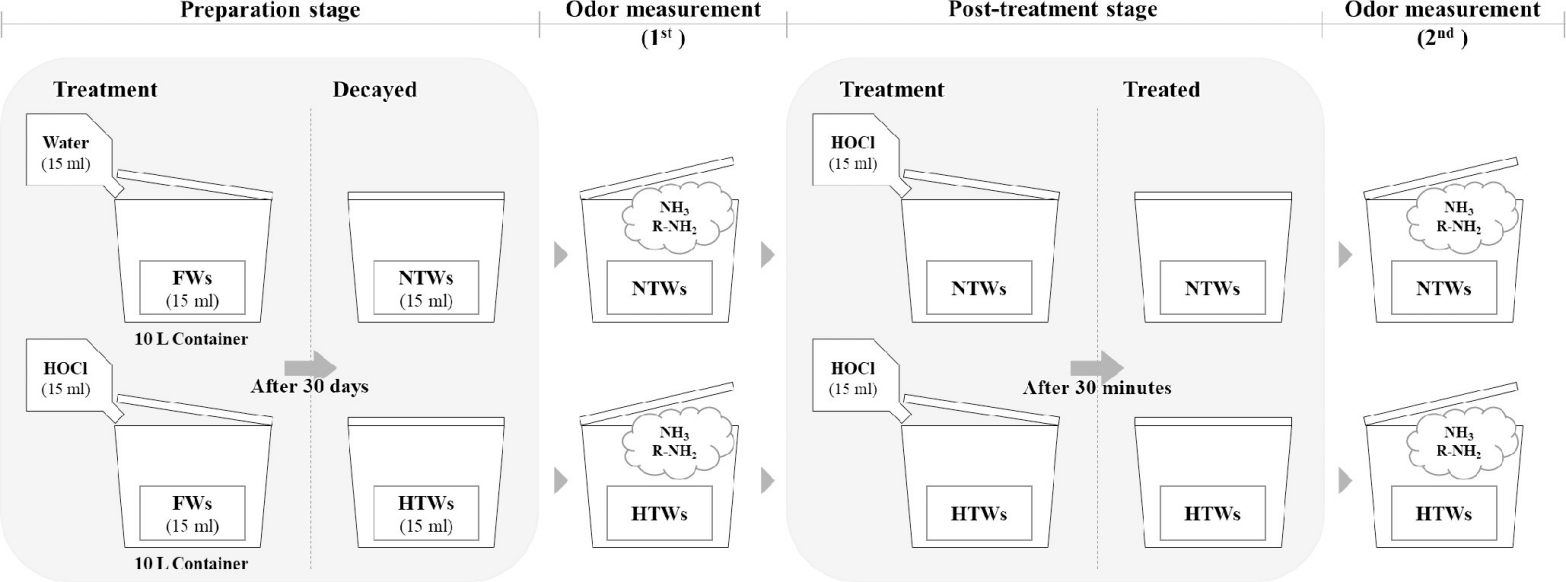
The experimental process to evaluate the deodorization effects of HOCl. Ammonia (NH_3_) and amine (R-NH_2_) gases are major components and sources of odor: food waste (FW), non-treated food waste (NTW), and HOCl-treated food waste (HTW).

To verify the odor suppression effect of HOCl, the acid was sprayed onto the HTW and NTW samples after measuring the gas concentration. The concentrations of NH_3_ and R-NH_2_ were measured again after 30 minutes. Then, the treatments, fermentation, and HOCl application were compared. In the odor suppression test, an earthworm was not introduced to isolate the effects of HOCl application on odor (Fig 3).

### Analytical methods and statistical analysis

The population size and individual weights of earthworms among the three groups were observed at the beginning and the end of experiment of earthworm growth test. They were compared using a non-parametric test (Kruskal-Wallis test) since the variables are non-normal. The Mann-Whitney test was also used for the post-hoc pair wise comparison when a significant difference was found among the groups.

## Results and discussion

### Test of the effectiveness for earthworm population growth

Table 1 summarizes the effectiveness of the HOCl application on the earthworm population and weight. The results of this experiment showed that the number of earthworms and total weight of the earthworms increased in all the breeding bins over the 60 days. Compared to the control (group 1), the HOCl application groups (group 2 and group 3) showed higher growth rates in both population and total weight. Group 3 with HOCl applications for moisture control and feed preparation turned out to be the most effective for earthworm reproduction and growth.

**Table 1:** Summary of the effectiveness of HOCI application by group within the vermicomposting period (60 days).

Categories		Control (Group 1)		Group 2		Group 3	
		Bin 1–1	Bin 1–2	Bin 2–1	Bin 2–2	Bin 3–1	Bin 3–2
Population (worms)	Day 0	20	20	20	20	20	20
	Day 60	75	76	95	103	118	129
	Difference (%)	**55 (▲275)**	**56 (▲280)**	**75 (▲375)**	**83 (▲415)**	**98 (▲490)**	**109 (▲545)**
Average Weight (g)	Day 0	0.497	0.470	0.505	0.510	0.473	0.501
	Day 60	0.255	0.279	0.306	0.276	0.312	0.259
	Difference (%)	**-0.242 (▽49)**	**-0.191 (▽41)**	**-0.199 (▽39)**	**-0.234 (▽46)**	**-0.161 (▽34)**	**-0.242 (▽48)**
Standard deviation (g)	Day 0	0.091	0.171	0.160	0.224	0.131	0.138
	Day 60	0.171	0.248	0.230	0.229	0.212	0.217
Total weight (g)	Day 0	9.937	9.396	10.100	10.193	9.450	10.026
	Day 60	19.134	21.218	29.068	28.435	36.771	33.395
	Difference (%)	**9.197 (▲93)**	**11.822 (▲126)**	**18.968 (▲188)**	**18.242 (▲179)**	**27.321 (▲289)**	**23.369 (▲233)**

Group 1 is the control group without any HOCl application. For group 2, HOCl (500 ml) was applied to food waste for feed preparation. For group 3, HOCl was used for both moisture control and feed preparation.

This study also compared the difference in the growth (population and individual weight) of earthworms among the three groups (N = 3) (see Table 2). Group 3 increased the population in the two breeding bins by 64% and 25%, respectively. The earthworm population after 60 days of breeding in group 3 was 57% to 72% greater than the population in group 1, and 24% to 35% greater than that in group 2.

**Table 2:** Results of the Kruskal-Wallis test and Mann-Whitney test conducted to confirm the effectiveness of the HOCl applications (a significance level (α) of 0.05).

	Test Type	Test Results	
		Individual Weight	Population
Group 1, 2, and 3	Kruskal-Wallis	p = 0.62 > α	0.00 < α
Group 1 vs. Group 2	Mann-Whitney	0.38 > α	0.00 < α
Group 1 vs. Group 3	Mann-Whitney	0.39 > α	0.00 < α
Group 2 vs. Group 3	Mann-Whitney	0.93 > α	0.00 < α

Group 1 is the control without any HOCl application. Group 2 represents HOCl application only on feed preparation. Group 3 represents HOCl application to both feed preparation and moisture control.

The total weight of the earthworms also increased during the experimental period. Similar to the increased population, the total weight of the two bins in group 3 increased by 233% and 289%, respectively (Table 1). However, the average weight of individual earthworms decreased over the experiment period at the similar rate in all three groups. These changes in population size and individual weight were evidently attributed to the number of newly hatched juveniles, and its effect was greater in the HOCl treated croups, in particular group 3. HOCl application is proposed to be a key contributor to the population growth by providing favorable conditions for reproduction.

Our finding indicates that HOCl application improved the general living conditions for the worms both directly and indirectly. HOCl is known to suppress the growth and development of some common pathogenic microbes in soil via enzymatic activities [43–46]. Additionally, vermicomposting worm species could maintain healthy microbial diversity in the soil by either consuming the soil microorganisms or nurturing a selective group of microbiota internally and externally [47]. The chloramination effect of HOCl might facilitate this direct effect of the earthworm on controlling the soil microorganisms by suppressing the harmful activity of common pathogenic bacteria in soil, as evidenced by the reduced production of nitrogen-metabolite gases.

The worm species, *Eisenia fetida*, is also known to inherit at least one gut bacteria species from its parents via egg capsules [48], and the gut bacteria are crucial factors in the vermicomposting process. Thus, it is plausible to propose that the gut-microbial composition and their composting activity was not significantly affected by the microbicidal effects of HOCl in the compost substrate. In the meantime, even though HOCl might not directly add any new nutrients that contribute to the population growth of the worms, it was possible that the antimicrobial effect indirectly supported the vermicomposting process and the worm reproduction, at least during this experiment period (one generational length of the worm species). In the present study, however, the change in the soil microbial diversity or gut bacterial composition under the HOCl treatments was not a direct interest; therefore, further studies on this subject and the long-term effects are warranted.

Warman and AngLopez [6] observed that the weight and population of the earthworms in their study increased by 150% and 60%, respectively, when the earthworms were allowed to grow without any treatment for 90 days. Their growth rates were similar to the control in our study. However, the growth rate of groups 2 and 3 in this study outperformed their non-treatment experiment group in terms of earthworm weight and population, which confirms the meaningful effectiveness of HOCl applications for earthworm growth.

### Test of effectiveness for odor suppression

The performance of HOCl for odor reduction is presented in Tables 3 and 4. The average concentrations of NH_3_ and R-NH_2_ in decayed food waste, as measured from the HTW treatment, were 15 ppm and 6 ppm, respectively. The average concentrations of NH_3_ and R-NH_2_ in NTW were 85 ppm and 10 ppm, respectively. The reduction effects of HTW were 82.3% and 40% compared to NTW.

**Table 3:** Effects (means ± S.E.) of HOCl pretreatment on the odor concentrations of food waste after decaying for 30 days (1st odor measurement): NTWs and HTWs indicate non-treated food waste and HOCl treated food waste, respectively.

Categories	NTWs		HTWs		Comparison (%)
	Before decay	Decayed	Before decay	Decayed	
NH_3_ (ppm)	0	85 ± 5	0	15 ± 5	82.3
R-NH_2_ (ppm)	0	10 ± 0	0	6 ± 1	40

**Table 4:** Effects (means ± S.E.) of continuous HOCl application on the odor concentrations of decayed food waste (2^nd^ odor measurement after 30 minutes): NTWs and HTWs indicate non-treated food waste and HOCl treated food waste, respectively.

Categories	NTWs			HTWs		
	Before application	After application	Comparison (%)	Before application	After application	Comparison (%)
NH_3_ (ppm)	70 ± 0	40 ± 5	42.9	18 ± 0	8.7 ± 5	51.7
R-NH_2_ (ppm)	10 ± 0	10 ± 0	0	7 ± 0	4.5 ± 1	35.8

Rayson et al. (2010) [49] and Tarade and Vrcek (2013) [50] showed that HOCl reacts with NH_3_ and R-NH_2_ and produces a chloramine substance, which is an effective disinfectant, depending on the ratio of chlorine to NH_3_ and R-NH_2_. The effective sterilization of HOCl could reduce the decay rate of food by disturbing the micro-organisms [51]. These results demonstrate that HOCl could suppress odor since HOCl changes the number of spoilage micro-organisms in the food waste and modifies the odor-causing substances [52].

The continuous application of HOCl reduced the odor of decayed food waste (Table 4). In particular, the results showed that HOCl was more effective with NH_3_ than R-NH_2_ (Table 4). Such result is attributed to the fact that the chlorine produced by the reaction with HOCl has the greater chloramination effect on NH_3_ than on R-NH_2_ [53]. The difference in the ammonia removal effect was also investigated because of the concentration difference in the NH_3_ gas presented at the beginning.

## Conclusion

This study investigated how HOCl applications can influence earthworm population growth and odor in small-scale indoor vermicomposting. The breeding bin tests showed that the application of HOCl on food waste for feed could promote earthworm reproduction and could reduce the odor represented by the ammonia (NH_3_) and amine (R-NH_2_) gas concentrations in the waste. Increased earthworm reproduction after HOCl application enhanced vermicomposting process. Moreover, chloramine created by the reaction between HOCl and the two odor gases was effective in removing NH_3_ than R-NH_2_. This study experimentally demonstrated that the HOCl application can improve the living condition for the earthworm and alleviate odor from food waste. The findings of this study are expected to help improve the efficiency and applicability of vermicomposting, especially in small-scale indoor conditions.
